# The Thyroid Status of Children and Adolescents in Fukushima Prefecture Examined during 20–30 Months after the Fukushima Nuclear Power Plant Disaster: A Cross-Sectional, Observational Study

**DOI:** 10.1371/journal.pone.0113804

**Published:** 2014-12-04

**Authors:** Hajime Watanobe, Tomoyuki Furutani, Masahiko Nihei, Yu Sakuma, Rie Yanai, Miyuki Takahashi, Hideo Sato, Fumihiko Sagawa

**Affiliations:** 1 Radiation Countermeasures Research Institute for Earthquake Disaster Recovery Support, Hirata, Fukushima, Japan; 2 Kasukabe Kousei Hospital, Kasukabe, Saitama, Japan; 3 Faculty of Policy Management, Keio University, Fujisawa, Kanagawa, Japan; 4 Hirata Central Clinic, Hirata, Fukushima, Japan; 5 Hirata Central Hospital, Hirata, Fukushima, Japan; Central South University, China

## Abstract

**Background:**

A possible increase in thyroid cancer in the young represents the most critical health problem to be considered after the nuclear accident in Fukushima, Japan (March 2011), which is an important lesson from the Chernobyl disaster (April 1986). Although it was reported that childhood thyroid cancer had started to increase 3–5 yr after the Chernobyl accident, we speculate that the actual period of latency might have been shorter than reported, considering the delay in initiating thyroid surveillance in the then Soviet Union and also the lower quality of ultrasonographic testing in the 1980s. Our primary objectives in the present study were to identify any possible thyroid abnormality in young Fukushima citizens at a relatively early timepoint (20–30 months) after the accident, and also to strive to find a possible relationship among thyroid ultrasonographic findings, thyroid-relevant biochemical markers, and iodine-131 ground deposition in the locations of residence where they stayed during very early days after the accident.

**Methods and Findings:**

This is a cross-sectional study. We targeted the Fukushima residents who were 18 yr old or younger (including fetuses) at the time of the accident. Our examinations comprised a questionnaire, thyroid ultrasonography, thyroid-related blood tests, and urinary iodine measurement. We analyzed a possible relationship among thyroid ultrasonographic findings (1,137 subjects), serum hormonal data (731 subjects), urinary iodine concentrations (770 subjects), and iodine-131 ground deposition (1,137 subjects). We did not find any significant relationship among these indicators, and no participant was diagnosed to contract thyroid cancer.

**Conclusions:**

At the timepoint of 20–30 months after the accident, we did not confirm any discernible deleterious effects of the emitted radioactivity on the thyroid of young Fukushima residents. This is the first report in English detailing the thyroid status of young Fukushima residents after the nuclear disaster.

## Introduction

A great earthquake of 9.0-Richter magnitude (Great East Japan Earthquake) struck the Pacific coast of Japan on March 11^th^ 2011. A subsequent tsunami severely damaged the cooling systems of the Fukushima Daiichi nuclear power plant (FNPP1), and a resultant hydrogen explosion caused the release of a large amount of radioactive material into the environment over March 2011. It was reported that iodine-131 (^131^I), cesium-134, and cesium-137 constituted a major proportion of the radioactivity discharged from the FNPP1 [1]. According to the report from the Japan Atomic Energy Agency, in the early afternoon of March 15^th^ a highly radioactive plume mainly arrived in such residential quarters that are located to the west and northwest of the crippled FNPP1 [2]. Thereafter, from the late afternoon of March 15^th^ to the early morning of March 16^th^ a continual rainfall occurred especially in the northern area of Fukushima Prefecture. It is considered that this rainfall lasting for about half a day deposited the atmospheric radionuclides including ^131^I on the ground of Fukushima Prefecture, and this constituted the majority of radiocontamination in the prefecture [2], [3].

This accident in Fukushima created a genuine concern about the human health risks that might be caused by radiation exposure. The most crucial health problem to be considered on the occasion of nuclear accidents is a possible increase in thyroid cancer in the young as a consequence of radioiodine taken up by the thyroid, because childhood thyroid cancer is the only malady that was accepted as the indubitable consequence of the Chernobyl nuclear disaster that occurred on April 26^th^ 1986 [4]. A scientific agreement has been reached that the age at radiation exposure is one of the most important modifiers of thyroid cancer risk [4]. From October 2011, as part of the Fukushima Health Management Survey the Fukushima Prefecture started periodic thyroid ultrasonographic surveillance for approximately 360,000 citizens in the prefecture who all were 18 yr old or younger at the time of the nuclear accident [5]. Their study protocol comprises thyroid ultrasonography as a primary examination, and thyroid-related blood testing and urinary iodine concentrations (UIC) as a second-stage examination [5]. The rationale for the UIC measurement is based on the fact that iodine deficiency serves as a significant potentiator of radiogenic thyroid cancer risk in the young [6]. According to the latest data declared by Fukushima Prefecture in August 2014 (3 yr and 5 months after the FNPP1 accident), the prefecture has thus far detected 104 cases of confirmed or suspected thyroid cancer among a total of 295,689 children and adolescents examined [7]. However, Fukushima Prefecture has yet to report the results of detailed analyses that are to be made of a possible relationship between thyroid ultrasonographic findings and individual thyroid exposure doses to ^131^I or the differential distribution of ground ^131^I contamination across Fukushima Prefecture.

Targeting the same age bracket of Fukushima residents as examined by Fukushima Prefecture, we also commenced thyroid examinations from November 2012 independently of the prefecture. Our thyroid examinations comprised a questionnaire (including an inquiry whether prophylactic stable iodine was taken), ultrasonography, and UIC measurement for all participants, and thyroid-relevant blood tests for those aged 6 yr or older at the time of the examinations, which as a whole constituted a more detailed study protocol than that by Fukushima Prefecture.

It was reported that childhood thyroid cancer had started to increase 3–5 yr after the Chernobyl accident [4], [8], [9]. However, because of the following reasons, we believe it still possible that after the Chernobyl disaster the number of patients with thyroid cancer might have started to rise at an earlier date than reported. First, it is almost certain that the authorities of the then Soviet Union had not envisaged the outbreak of childhood thyroid cancer until the tragedy became obvious, which caused a delay in initiating sufficient surveillance of the young thyroid. Second, considering the fact that neck ultrasonography came into play in the 1980s, the imaging quality of ultrasound machines employed for thyroid checkups during an early stage after the Chernobyl accident, must have been lower than that in the 2010s.

Therefore, in this study we aimed to conduct the following in more than 1,000 children and adolescents in Fukushima Prefecture: (1) to identify any possible abnormality in the thyroid by ultrasonography at a relatively early timepoint (20–30 months) after the accident, and (2) to strive to find any possible relationship among thyroid ultrasonographic findings, the data of thyroid-related blood tests and UIC, and also ^131^I ground deposition in the locations of residence where individual participants stayed during a very early period after the FNPP1 accident.

## Methods

### Ethics statement

This study was reviewed and approved by the ethical committee of the Radiation Countermeasures Research Institute for Earthquake Disaster Recovery Support (http://www.fukkousien-zaidan.net), and all participants (or their legal guardians) signed an informed consent form. A separate written informed consent was obtained for fine-needle aspiration biopsy of the thyroid (see “ultrasound examination” below). This study was conducted according to the principles of the Helsinki Declaration.

### Study participants

A total of 1,222 subjects (614 males, 608 females), who all were 18 yr old or younger (including fetuses) at the time of the FNPP1 accident, participated in this study between November 10^th^ 2012 and September 30^th^ 2013 (20–30 months after the accident) from all across Fukushima Prefecture. Our examinations comprised a questionnaire (for all participants), thyroid ultrasonography (for all participants), thyroid-related blood tests (for the subjects aged 6 yr or older at the examinations unless they declined), and UIC measurement (for all participants unless they declined). All these examinations were done on the same day.

### Questionnaire

All participants or their legal guardians were invited to fill out a questionnaire that addressed personal and family histories of any disease (including thyroid disorders), whether and when they took potassium iodide (KI) distributed by Fukushima Prefecture immediately after the hydrogen explosion at the FNPP1 Unit 1 on March 12^th^ 2011 [2], whether they increased the ingestion of seaweed following the explosion, whether and when they evacuated inside or outside Fukushima after the accident, and how long they continued staying in the same area within Fukushima on and after March 15^th^ 2011 (the date when the majority of the radiocontamination occurred in Fukushima). This final inquiry was not applicable to the subjects who evacuated outside Fukushima. As to KI, Fukushima Prefecture recommended that KI be taken upon instructions from the prefecture at different doses in different age groups in accordance with the WHO's Guidelines for Iodine Prophylaxis Following Nuclear Accidents [10].

### Ultrasound examination

All subjects underwent thyroid ultrasonography using a 9.0-MHz linear transducer (LOGIQ S8; Yokogawa GE Medical Systems, Ltd., Tokyo, Japan) by certified ultrasonographers. Presence of nodules, echostructure, and pattern of echogenicity were recorded as per the guidelines for the diagnosis and treatment of thyroid tumors publicized by the Japan Society of Clinical Oncology [11]. Thyroid nodules, if detected, were classified into solid nodules or cysts, and their size and location were recorded. A cystic nodule with a solid component was classified as a solid nodule. Special caution was exercised to differentiate congenital aberrations in the thyroid (ectopic thymus and ultimobranchial body) from solid nodule or cyst. Nodule diameter was measured in three dimensions and recorded to the nearest 0.1 millimeter. All the recorded films were reviewed by a certified endocrinologist specializing in thyroid diseases. Participants who were suspected of having a malignant solid nodule were recommended to undergo echoguided fine-needle aspiration biopsy for cytological examinations.

### Serum assays

Blood samples were drawn for the measurement of thyroid-stimulating hormone (TSH), free triiodothyronine (fT_3_), free thyroxine (fT_4_), thyroglobulin (Tg), anti-thyroglobulin antibody (TgAb), and anti-thyroid peroxidase antibody (TPOAb). TSH, fT_3_, and fT_4_ were measured by immunochemiluminescence assay utilizing the kits produced by Siemens Healthcare Diagnostics Inc. (Los Angeles, CA, USA). Tg, TgAb, and TPOAb were measured by electro-chemiluminescence immunoassay utilizing the kits produced by Roche Diagnostics International Ltd. (Rotkreuz, Switzerland). All assays were done in duplicate. The analytical sensitivities and normal ranges (in parentheses) of the assays for TSH, fT_3_, fT_4_, Tg, TgAb, and TPOAb were 0.008 mIU/L (0.4–4.0), 0.31 pmol/L (3.4–6.3), 1.3 pmol/L (10.3–24.5), 0.1 µg/L (<32.7), 10 kIU/L (<28.0), and 5 kIU/L (<16.0), respectively. In all the six assays, both the inter- and intraassay coefficients of variation were less than 10%.

### Urinary iodine measurement

The UIC was determined in the spot urine by the Sandell-Kolthoff reaction utilizing the kit produced by Hitachi Chemical Co., Ltd. (Tokyo, Japan). All urine samples were assayed in duplicate. The sensitivity of the assay was 25 µg/L, and both the inter- and intraassay coefficients of variation were less than 10%.

### ^131^I ground deposition

**Figure 1:**
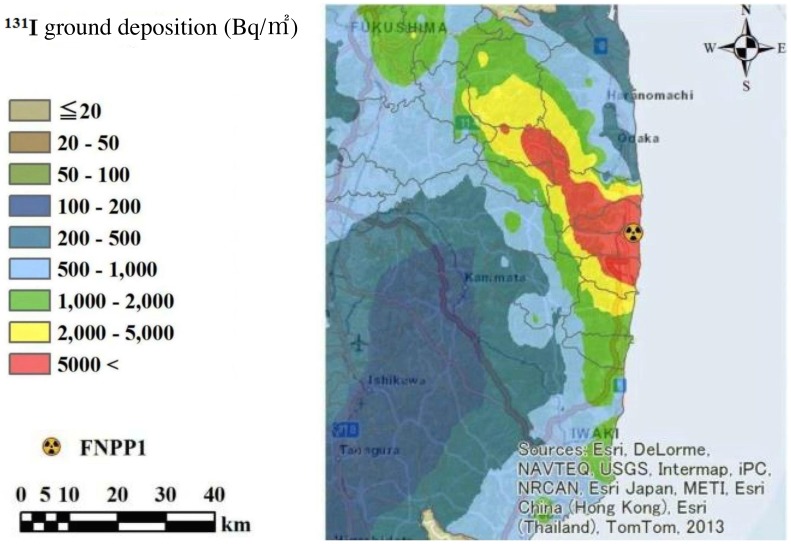
Estimated ^131^I ground deposition across Fukushima Prefecture as of March 15^th^ 2011. FNPP1: Fukushima Daiichi Nuclear Power Plant.

Only limited information had been available as to ^131^I ground deposition in Fukushima Prefecture at early days after the FNPP1 accident, until Torii *et al.* [12] reported data pertaining to it. However, the geographical distribution of ^131^I described in this previous study did not encompass all areas of Fukushima Prefecture. Thus, we constructed a map of estimated ground deposition of ^131^I all across Fukushima Prefecture based on the data reported elsewhere [13], [14], employing empirical Bayesian kriging interpolation method [15]. In the estimated map (Figure 1) the radioactivity of ^131^I is decay-corrected as of March 15^th^ 2011, at which time the majority of radioiodine was considered to have been deposited in Fukushima Prefecture [2], [3]. The absolute values of deposited ^131^I in Figure 1 are similar to the levels reported in other studies [12], [16].

### Statistical analysis

Results were expressed as percentage, the mean ± S.D., or the median (IQR). To compare data among different groups, Chi-square test, pairwise *t*-test, analysis of variance (ANOVA), Kruskal-Wallis test, or non-parametric multiple comparison test was used as appropriate. Correlation among variables was tested using regression analysis. Intergroup differences and correlation were considered significant if *P* was smaller than 0.05.

## Results

### Questionnaire and sample sifting processes towards analyses

**Figure 2:**
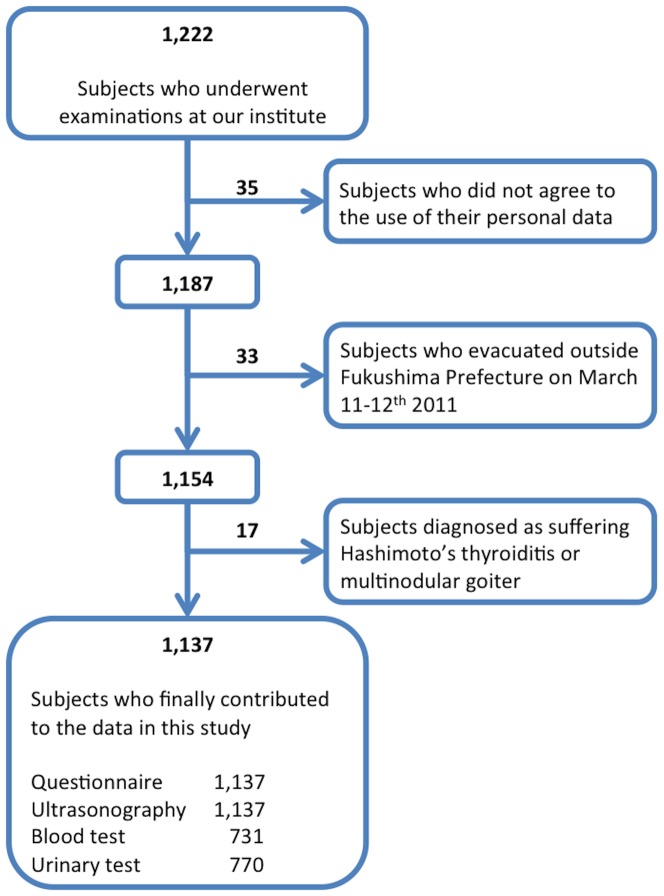
Sample sifting processes towards analyses.

Thirty-five (2.9%) of the 1,222 subjects did not agree that their personal data would be used for analyses, so that the results from these participants were not subjected to subsequent analyses (Figure 2). The questionnaire revealed that all participants had lived somewhere in Fukushima Prefecture before the earthquake broke out, but 19.9% (236 of 1,187) of them evacuated their hometown as per the instructions declared by the Japanese authorities on March 11–12^th^ 2011 for the residents living within a radius of 20 km from the crippled FNPP1 [17]. Of these evacuees, 203 people evacuated within and 33 people outside Fukushima Prefecture. Data from these 33 people were not subjected to statistical analyses in this study, because for this sample population we did not have data on ^131^I ground deposition that was integral for our analyses (Figure 2). From the questionnaire, we also learned that most of the 951 non-evacuated and 203 evacuated (inside Fukushima) subjects had stayed in the same domicile from March 15^th^ to 31^st^ 2011 (for 17 days). This 17-day period was such a length of time by the end of which the radioactivity of ^131^I, a gamma and beta emitter with a physical half-life of 8 days, must have decayed down to about 23% of that released on March 15^th^ 2011. In subsequent analyses involving ^131^I ground deposition, each participant's domicile during these 17 days was employed to apply area-specific levels of deposited ^131^I to each individual. In a small proportion of the participants who changed their domiciles during these 17 days, the areas where such individuals stayed for the longest period of time were employed to allocate to them individual levels of ^131^I ground deposition.

Of these 1,154 subjects, 17 did not contribute to statistical analyses because of their ultrasonographic and/or hormonal data (Figure 2). Fifteen (two males, 13 females) of the 17 participants were diagnosed as Hashimoto's thyroiditis, and the remaining two females as multinodular goiter as per the diagnostic criteria for the two diseases [18]. It is well established that TgAb is positive in the majority of the patients with Hashimoto's thyroiditis (in fact positive in all our 15 patients), and also known to interfere with the measurement of Tg [19], which is an analytical parameter in the current study. Another reason for excluding the patients with Hashimoto's thyroiditis and multinodular goiter from statistical analyses is that both disorders are pathogenetically irrelevant to radiation exposure [18]. We conducted fine-needle aspiration biopsy for potentially malignant solid nodules in two subjects, and in both of them the nodule cytologically proved benign.

**Figure 3:**
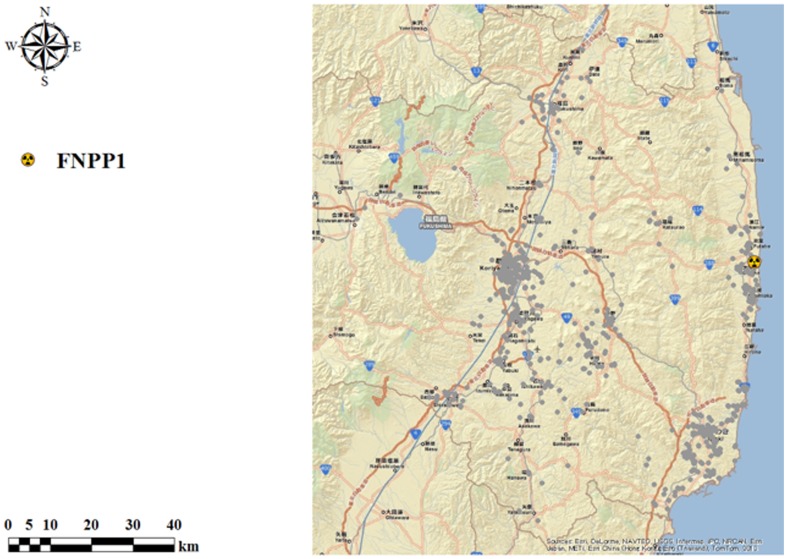
Individual locations of residence where the final 1,137 subjects stayed during the period of March 15^th^–31^st^ 2011. The dots indicate the individual locations of residence. FNPP1: Fukushima Daiichi Nuclear Power Plant.

Collectively, in the present study the questionnaire and ultrasonographic data from 1,137 subjects [578 males, 559 females; mean (± S.D.) age, 6.1±4.6 yr], blood test results from 731 subjects (374 males, 357 females), and UIC data from 770 participants (392 males, 378 females) contributed to the analyzed results given below (Figure 2). The questionnaire revealed that among this final population 4.0% (45 of 1,137) of the subjects took KI, and in each of them KI was taken only one time between 2:00 p.m. March 14^th^ and 10:00 p.m. March 15^th^ 2011. This timing of KI intake seemed appropriate considering a previous report that KI is effective to reduce thyroid uptake of radioiodine only when it was taken between 48 h before and 8 h after exposure to radioiodine [20]. In addition, we learned that 23.1% (263 of 1,137) of the participants increased seaweed consumption after the hydrogen explosion on March 12^th^ 2011, and 0.4% (5 of 1,137) of the subjects both took KI and increased seaweed ingestion. We also learned from the questionnaire that every participant had kept up their dietary habits on and after March 12^th^ 2011 until they underwent examinations in this study. Figure 3 shows the individual locations of residence where these final 1,137 subjects stayed during the period of March 15^th^–31^st^ 2011.

### Effects of prophylactic deeds against thyroid exposure to ^131^I

In the first place, we compared the two groups with and without the prophylactic deeds against thyroid exposure to ^131^I (KI intake and increased seaweed consumption after the accident). As shown in Table 1, it was revealed that the prophylactic deeds did not significantly affect the prevalence, number, or the largest diameter of either solid nodule or cyst in the thyroid, serum hormonal data, or UIC. Therefore, the data from these two sample populations were combined together for all subsequent analyses.

**Table 1:** Comparison of thyroid ultrasonographic findings, serum hormonal data, and urinary iodine concentrations between the two groups with and without prophylactic actions against thyroid exposure to radioiodine after the nuclear accident.

	Number of subjects	Prevalence of nodule^a^ (%)	Preva-lence of solid nodule (%)	Preva-lence of cyst (%)	Total number of nodules^b^^,^ c	Number of solid nodules^c^	Number of cysts^c^	The largest diameter of solid nodule^d^ (mm)	The largest diameter of cyst^d^ (mm)	Serum TSH (mIU/L)^e^	Serum fT_3_ (pmol/L)^e^	Serum fT_4_ (pmol/L)^e^	Serum Tg (µg/L)^e^	UIC (µg/L)^f^
**Group I**	303	74.1	4.1	72.5	2.5±1.6^g^	0.06±0.33	2.4±1.6	5.1±3.1	2.5±1.0	2.0±1.0 (208)^i^	6.3±0.7 (208)	16.5±1.9 (208)	22.4±21.2 (208)	294 (414)^h^ (232)
**Group II**	834	72.4	4.2	70.9	2.4±1.7	0.05±0.23	2.4±1·7	5.2±3·0	2.6±1.1	2.0±1.1 (523)	6.3±0.8 (523)	16.4±1.9 (523)	20.9±12·3 (523)	262 (377) (538)

Group I comprises the subjects who took potassium iodide (KI) after the nuclear accident, those who increased seaweed consumption following the accident, and those who did both.

Group II comprises the subjects except Group I.

a Percentage of subjects who had solid nodules, cysts, or both.

b The sum of the numbers of solid nodules and cysts in each subject.

c The number was calculated including the subjects without solid nodule or cyst, who were considered to have a “zero” lesion.

d The data were derived from only the subjects who had solid nodules (47 subjects) or cysts (811 subjects).

e Serum hormonal data were derived from a total of 731 subjects.

f UIC data were derived from a total of 770 subjects.

g Mean ± S.D.

h Median (IQR).

i The numbers in parentheses indicate the number of subjects who contributed to the serum hormonal or UIC data in the respective groups.

Differences between the two groups were analyzed by Chi-square test (for the percentage data), pairwise *t*-test (for the mean ± S.D. data), or Kruskal-Wallis test [for the median (IQR) data].

There were no statistically significant differences in the thyroid ultrasonographic findings, serum hormonal data, and the UIC between the two groups.

TSH, thyroid-stimulating hormone; fT_3_, free triiodothyronine; fT_4_, free thyroxine; Tg, thyroglobulin; UIC, urinary iodine concentration.

### Relationship between thyroid nodularity and ^131^I ground deposition

**Figure 4:**
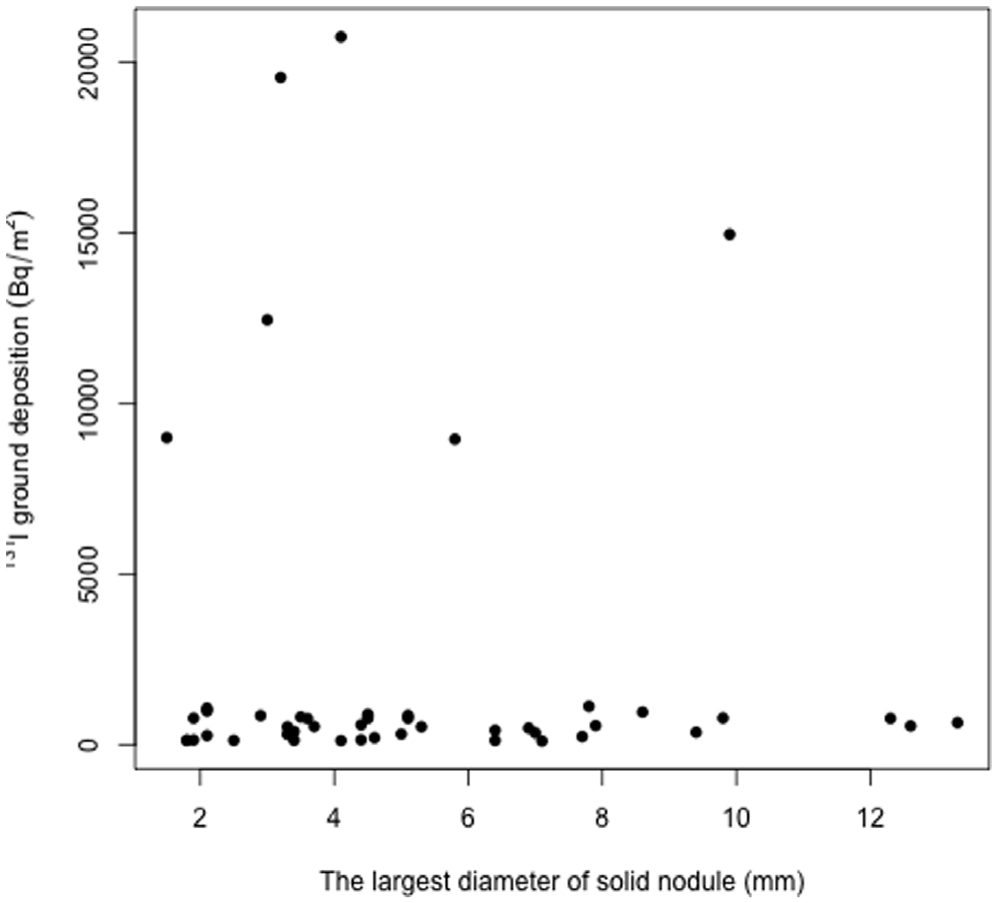
Scatter plot of the largest diameter of solid nodule *vs.* the ^131^I ground deposition in the 47 subjects who had solid nodules in the thyroid. Regression analysis revealed that there was no significant correlation between the two parameters.

**Figure 5:**
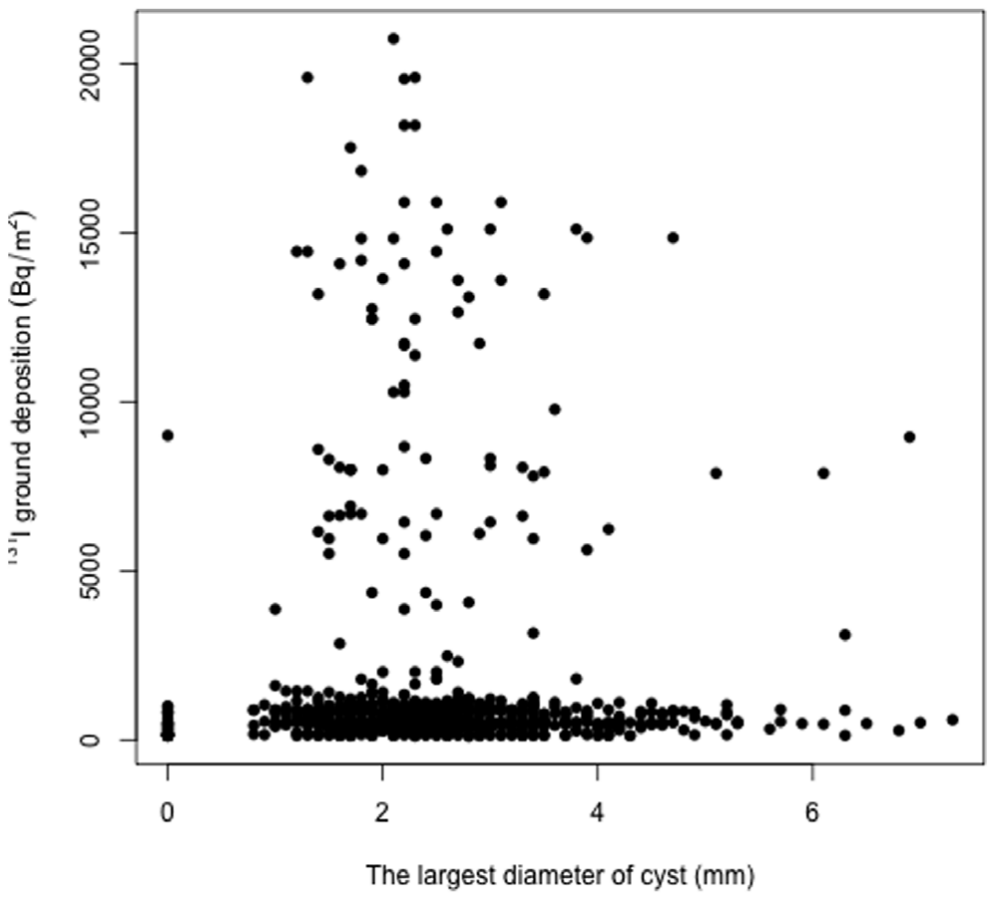
Scatter plot of the largest diameter of cyst *vs.* the ^131^I ground deposition in the 811 subjects who had cysts in the thyroid. Regression analysis revealed that there was no significant correlation between the two parameters.

We analyzed whether the total number of nodules (solid nodules and cysts combined) and the number of solid nodules or cysts had any significant relationship with the ^131^I ground deposition in the locations of residence where individual participants stayed during the early days after the nuclear accident. As shown in Table 2, the deposited ^131^I levels were slightly (1.2-fold) but significantly (*P* = 0.007) higher in the “three-nodule” group than in the “zero-nodule” group. Similarly, the “three-cyst” group had a slightly (1.2-fold) but significantly (*P* = 0.004) higher level of deposited ^131^I than the “zero-cyst” group. The number of solid nodules was not significantly related to the deposited levels of ^131^I. We also analyzed whether the largest diameter of solid nodules (detected in 47 subjects) and cysts (detected in 811 subjects) had any significant correlation with ^131^I ground deposition, and found that neither of these thyroid indicators was significantly correlated with the extent of ^131^I contamination on the ground (Figures 4 and 5).

**Table 2:** Relationship between the thyroid nodularity and the ^131^I ground deposition in the locations of residence after the nuclear accident.

	Number of lesions	Number of subjects	^131^I ground deposition in the locations of residence (Bq/m^2^)^a^
**Total number of nodules** b	0	308	517 (482)^c^
	1	122	525 (539)
	2	98	578 (552)
	3	174	630 (513)^d^
	4 or more	435	536 (499)
**Number of solid nodules**	0	1,090	532 (498)
	1	41	601 (541)
	2	5	286 (12,245)
	3 or more	1	578 (0)
**Number of cysts**	0	326	517 (486)
	1	121	526 (569)
	2	85	568 (535)
	3	174	635 (506)^e^
	4 or more	431	535 (500)

a Values of individual subjects were determined based on the data shown in Figure 1.

b The sum of the numbers of solid nodules and cysts in each subject.

c Median (IQR).

Non-parametric multiple comparison test with Kruskal-Wallis test was employed to analyze whether the ^131^I ground deposition differed as a function of the total number of nodules and the number of solid nodules or cysts.

d Significantly different from the “zero-nodule” group (*P* = 0.007).

e Significantly different from the “zero-cyst” group (*P* = 0.004).

### Relationship between the UIC and thyroid ultrasonographic and hormonal data

We analyzed whether the thyroid nodularity and thyroid-relevant hormonal data altered as a function of the UIC. As per the WHO criteria for the UIC [21], we compared various indicators of thyroid nodularity and thyroid-related hormonal data across five subgroups that comprised “moderate to severe iodine deficiency” (<49 µg/L), “mild iodine deficiency” (50–99 µg/L), “adequate iodine intake” (100–199 µg/L), “more than adequate iodine intake” (200–299 µg/L), and “excessive iodine intake” (>300 µg/L). As shown in Table 3, thyroid nodularity and hormonal data were statistically indistinguishable among the five subgroups.

**Table 3:** Thyroid ultrasonographic findings and thyroid-related hormonal data in the five groups with different ranges of urinary iodine concentrations.

UIC (µg/L)	∼49	50∼99	100∼199	200∼299	300∼
Number of subjects	49	119	248	128	226
Total number of nodules^a^^,^ b	2.5±1.7	2.6±1.5	2.7±1.5	2.6±1.5	2.5±1.6
Number of solid nodules^b^	0.04±0.24	0.08±0.42	0.05±0.20	0.04±0.15	0.04±0.23
Number of cysts^b^	2.5±1.7	2.6±1.5	2.7±1.5	2.6±1.5	2.4±1.6
The largest diameter of solid nodule (mm)^c^	1.5^d^	5.8±2.7	5.7±3.6	3.7±0.7	4.9±2.0
The largest diameter of cyst (mm)^c^	2.6±0.9	2.6±1.1	2.6±1.1	2.4±0.8	2.4±1.0
Serum TSH (mIU/L)^e^	2.3±1.3 (30)^f^	2.0±1.0 (85)	1.9±1.2 (177)	1.9±0.9 (89)	1.9±1.0 (147)
Serum fT_3_ (pmol/L)^e^	6.3±1.6 (30)	6.3±0.8 (85)	6.3±0.9 (177)	6.2±0.6 (89)	6.3±0.6 (147)
Serum fT_4_ (pmol/L)^e^	15.5±1.3 (30)	15.5±1.5 (85)	16.8±2.6 (177)	16.8±2.1 (89)	16.6±1.7 (147)
Serum Tg (µg/L)^e^	22.7±10.3 (30)	24.0±28.2 (85)	23.8±24.0 (177)	20.8±11.1 (89)	22.8±14.1 (147)

Data are expressed as the mean ± S.D.

a The sum of the numbers of solid nodules and cysts in each subject.

b The number was calculated including the subjects without solid nodule or cyst, who were considered to have a “zero” lesion.

c The data were derived from only the subjects who had solid nodules or cysts.

d In this UIC group, only one subject had a solid nodule.

e Serum hormonal data were derived from a total of 528 subjects.

f The numbers in parentheses indicate the number of subjects who contributed to the serum hormonal data in the respective groups.

Intergroup differences were analyzed by ANOVA, and there were no statistically significant differences in the ultrasonographic indicators and hormonal data among the five groups.

UIC, urinary iodine concentration; TSH, thyroid-stimulating hormone; fT_3_, free triiodothyronine; fT_4_, free thyroxine; Tg, thyroglobulin.

### Other analyses

We examined a possible relationship between the number and the largest diameter of solid nodules and cysts and the serum levels of TSH, fT_3_, fT_4_, and Tg, and found no significant relationship among these parameters (data not shown). Finally, in 46 subjects who were *in utero* at the time of the nuclear accident, we analyzed the relationship between thyroid nodularity and ^131^I ground deposition in the locations of residence where their mothers stayed between March 15^th^ and 31^st^ 2011. The result was that neither the number nor the largest diameter of their solid nodules and cysts was significantly related to the deposited levels of ^131^I (data not shown). Results of the questionnaire revealed that none of the mothers of these 46 children took KI or increased seaweed consumption after the accident.

## Discussion

In this study we examined the thyroid status of 1,137 children and adolescents in Fukushima Prefecture at the timepoint of 20–30 months after the FNPP1 accident. The results were that we did not detect any confirmed case of thyroid cancer in our sample population, and that we did not find any significant relationship among thyroid ultrasonographic findings, thyroid-relevant hormonal data and UIC. However, as discussed below, the total number of thyroid nodules and the number of cysts tended to be larger as the ^131^I ground deposition elevated.

The questionnaire in the current study revealed that after the nuclear accident in Fukushima 4.0% of the participants took KI, and 23.1% of the subjects increased seaweed consumption of their own will. Immediately after the hydrogen explosion on March 12^th^ 2011, Fukushima Prefecture distributed KI to the residents aged 40 yr or younger who lived within a radius of 50 km from the damaged FNPP1 so that they could take KI when instructed [22]. However, what proportion of them actually took KI was unknown [23], nor was included any inquiry about it in the questionnaire conducted by Fukushima Prefecture [5]. In this regard, the hard number of 4.0% that we obtained from our own questionnaire seems to be the first reliable data as to the proportion of young Fukushima residents who actually took KI after the nuclear accident. The other figure of 23.1%, a proportion of the participants who increased seaweed consumption after the accident, is also informative because this indicates that about one-fourth of the subjects and/or their parents knew that the seaweed is a foodstuff rich in natural iodine and the dietary iodine has protective effects on thyroid exposure to radioiodine. At any rate, we found in this study that neither KI intake nor changes in dietary habits significantly affected the thyroid nodularity of the participants at least at the timepoint of 20–30 months after the nuclear accident.

In the present study we found that the ^131^I ground deposition was slightly but significantly higher in the “three-nodule” group than in the “zero-nodule” group, and also in the “three-cyst” group compared to the “zero-cyst” group. Because the number of solid nodules was not significantly related to the ^131^I ground deposition, it is very likely that the significantly higher ^131^I levels in the “three-nodule” group reflected the similar predominance of deposited ^131^I levels in the “three-cyst” group over the “zero-cyst” group. However, because there was no dose-dependent relationship between the ^131^I ground deposition and the number of nodules or cysts, we do not interpret our present data as suggesting a cause-and-effect relationship between radioiodine exposure and thyroid cyst formation. Even so, further investigations along these lines would be interesting and also warranted.

In the atmosphere, iodine exists in both gaseous and particulate forms [24]. It was reported that after the Chernobyl accident the majority of ^129^I and ^131^I discharged had been observed in organic gaseous form [25], [26]. Based on these findings, it is very likely that after the FNPP1 accident inhalation served as the primary pathway of radioiodine uptake to the thyroid. Owing to methodological difficulties, we could not make a reliable estimate of individual thyroid equivalent doses, so that we employed the ^131^I ground deposition as a surrogate marker for the individual exposure to ^131^I. However, this is one of the limitations to the present study because the transfer of the deposited ^131^I to the thyroid represents a major dosimetric uncertainty. Notwithstanding these limitations, we believe that our present study is the first to have analyzed in the young Fukushima residents the relationship between the thyroid ultrasonographic findings and the differential levels of ^131^I ground deposition across Fukushima Prefecture. As of today, Fukushima Prefecture or any other institution has yet to report the results of studies conducted from the same standpoint. All the latest information publicized by Fukushima Prefecture as regards the exposed radiation dose in young Fukushima citizens is that the external exposure of radiation was able to be estimated in only 57 (0.019%) of 295,689 people aged 18 yr or younger at the time of the accident, and the dose was universally below 2.5 mSv [7]. However, this estimated dose was expressed as the effective dose, but not as the thyroid equivalent dose, and furthermore the calculation was based on the predicted external exposure to all radionuclides emitted by the FNPP1 accident, without being specified to ^131^I [7], [27].

A previous report that iodine deficiency is a significant potentiator of radiogenic thyroid cancer risk in the young [6] constitutes a rationale for us to compare thyroid nodularity across the subjects with various UIC, paying particular attention to the participants with subnormal UIC levels. In this study, we found that none of the indicators of thyroid nodularity was significantly related to the iodine status ranging from severe deficiency to excess intake. This negative finding was also the case with the relationship between the UIC and serum TSH, FT_3_, FT_4_, and Tg. However, it has been reported that Tg is a suitable indirect marker for iodine status in both adults and children, with higher Tg levels being observed under both deficiency and excess of iodine [28], [29]. We have no clear explanation for this apparent discrepancy, but the relatively small sample size in our investigation might have been responsible. Collectively, these results suggest that iodine status of the young Fukushima residents does not seem to affect their thyroid nodularity or function at least at the timepoint of 20–30 months after the nuclear accident, although this does not exclude the possibility of different results emerging in the foreseeable future. There is a limitation to our measurement of UIC. Although the UIC data in the present study provide information on recent iodine intake of the subjects, they do not necessarily reflect their iodine status at the time of the FNPP1 accident. Even so, because we learned from our questionnaire that every participant in this study kept up their dietary habits on and after March 12^th^ 2011 (the date when the hydrogen explosion broke out at the FNPP1 Unit 1) until they underwent examinations in the present study, it is very likely that the UIC data reported herein well reflected the iodine status of our participants back in the early days after the nuclear accident.

Information is very limited regarding the effects of *in utero* radiation exposure on thyroid cancer risks in postnatal life. Results from several previous studies on this issue are inconclusive [30]–[32]. In the present study we conducted thyroid ultrasonography on 46 subjects who might have been exposed to radioiodine *in utero*, and found no significant relationship between the thyroid nodularity of the children and the ^131^I ground deposition in the locations of residence where their mothers stayed during the very early days after the FNPP1 accident. Fukushima Prefecture commenced thyroid ultrasonography only from April 2014 on such residents who were *in utero* at the time of the accident [5], and thus our current study is the first to report the thyroid findings of Fukushima inhabitants who were fetuses then. However, because our data were derived from the small sample size and obtained during 20–30 months after the accident, further investigations are warranted to provide a more valid implication.

According to the latest data publicized by Fukushima Prefecture in August 2014 (3 yr and 5 months after the FNPP1 accident), the prefecture detected 104 cases of confirmed or suspected thyroid cancer among 295,689 Fukushima citizens who were 18 yr old or younger at the time of accident [7]. This prevalence (equivalent to 352 cases per million) is 29 times greater than the officially documented latest incidence of thyroid cancer (12 cases per million in the year 2010) among the Japanese people aged 19 yr or younger [33]. In the case of Chernobyl fallout, the annual incidence of thyroid cancer in Belarussian children in 1991–1992 (5–6 yr after the accident) was reported to be 62 times greater than that in the 10-yr period before the disaster [34]. It is uncertain at the present time whether the 29-fold rise in childhood thyroid cancer in Fukushima at the timepoint of about 3.5 yr after the accident represents a genuine increase or a consequence of the rigorous thyroid surveillance conducted by Fukushima Prefecture. In this regard, a caveat was very recently published that the current thyroid screening program implemented by Fukushima Prefecture might probably be lacking in power to fulfill the foremost objective of elucidating the health impact of radiation exposure, and simply be leading to the over-diagnosis and over-treatment of thyroid cancer in the young [35]. Along these lines, it is also true that there are no data available to date that can decidedly disprove the involvement of the FNPP1 accident in the pathogenesis of at least part, if any, of the above-mentioned 104 cases of confirmed or suspected thyroid cancer.

Chernobyl and Fukushima are similar in that they both are a nuclear reactor accident, but there are notable differences between the two in terms of the risk for thyroid cancer development as in the following: (1) The total amount of radioiodine emitted from the FNPP1 was as small as about one-tenth of that released after the Chernobyl accident [23]. Indeed, ^131^I ground deposition densities measured in Fukushima after the accident [12], [16, this study] were extremely small compared to those in the most contaminated areas in Ukraine after the Chernobyl accident [36]. (2) Whereas after the Chernobyl disaster the mean thyroid equivalent dose in evacuees was reported to be 490 mSv [4], the thyroid equivalent dose in Fukushima children was 44 mSv at maximum in one study [37] and a median dose was 4.2 mSv in another study [38]. (3) In the case of Chernobyl, neither food restriction nor KI distribution was in place in Belarus, the Ukraine, or the western regions of Russia, all of which were heavily contaminated by the accident [4], and therefore the ingestion of radioiodine-contaminated milk served as a major contributing factor to the epidemic of radiogenic thyroid cancer in infants [39]. By contrast, after the FNPP1 accident the Japanese authorities implemented fairly rigorous food safety regulations at its early stage [40]. (4) After the Chernobyl accident iodine deficiency played a significant role in increasing the risk of radiogenic thyroid cancer [6], while in the case of Fukushima a sufficient amount of natural iodine included in the traditional Japanese diet may probably have contributed to blocking radioiodine uptake by the thyroid [23]. With respect to the radiation doses for solid cancer induction, the United Nations Scientific Committee on the Effects of Atomic Radiation (UNSCEAR) reported that a statistically significant risk elevation is not observed at doses of 100 mSv or less [41]. However, it should also be remembered that no threshold dose is known for radiation-induced genetic effects and carcinogenesis [42].

In summary, this study examined the thyroid status of 1,137 children and adolescents in Fukushima Prefecture at the timepoint of 20–30 months after the FNPP1 accident. The results obtained revealed no discernible deleterious influences of the emitted radioactivity on the young thyroid. In addition, we did not find any significant relationship between the thyroid ultrasonographic findings and thyroid-relevant biochemical markers. Although the number of thyroid cysts tended to be larger as the ^131^I ground deposition elevated, the results obtained were not solid enough to suggest a cause-and-effect relationship between radioiodine exposure and thyroid cyst formation. We admit that the present study suffers two major methodological limitations, i.e., (1) We employed the differential levels of ^131^I ground deposition as a surrogate marker for individual thyroid exposure estimates, and (2) The UIC data in the current study do not necessarily reflect the participants' iodine status at the time of the nuclear accident. However, both of these limitations were insurmountable. Whether all the negative results in the current study suggest an insignificant health impact of the FNPP1 accident or reflect the relatively early implementation of thyroid examinations in the wake of the accident, must await forthcoming studies. Further continuing studies are warranted in terms of the long-term health surveillance and protection of the radiation-exposed children and adolescents in Fukushima. Although the sample size in our investigation was small, this is the first report in English detailing the thyroid status of young Fukushima residents after the nuclear disaster.
