# Correction: The Thyroid Status of Children and Adolescents in Fukushima Prefecture Examined during 20–30 Months after the Fukushima Nuclear Power Plant Disaster: A Cross-Sectional, Observational Study

**DOI:** 10.1371/journal.pone.0133345

**Published:** 2015-07-15

**Authors:** Hajime Watanobe, Tomoyuki Furutani, Masahiko Nihei, Yu Sakuma, Rie Yanai, Miyuki Takahashi, Hideo Sato, Fumihiko Sagawa

There are errors in the caption for Fig 1. Please see the complete, correct Fig 1 caption here.

**Fig 1:**
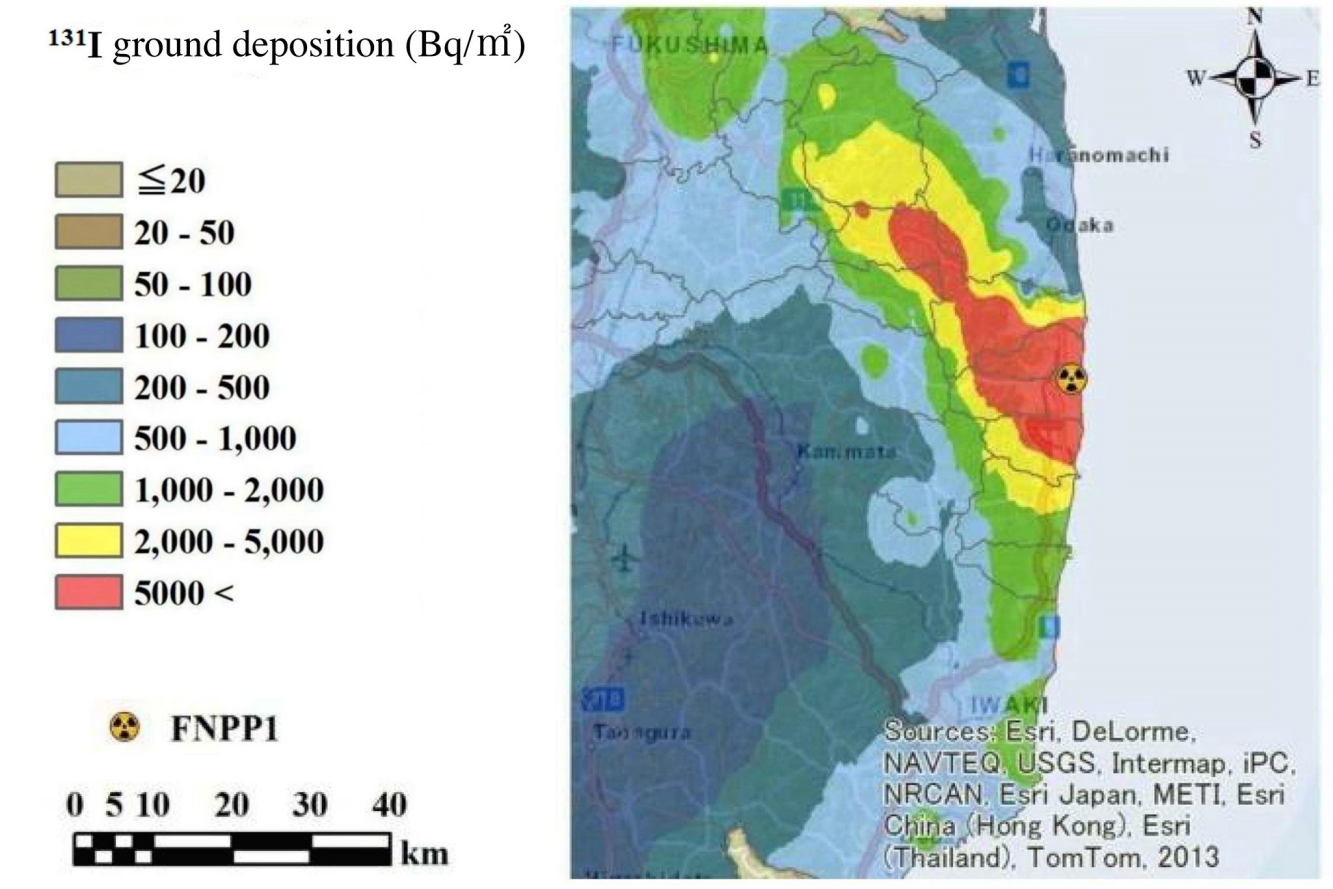
Estimated 131I ground deposition across Fukushima Prefecture as of June 14th 2011. FNPP1: Fukushima Daiichi Nuclear Power Plant.

## References

[1] Watanobe H, Furutani T, Nihei M, Sakuma Y, Yanai R, Takahashi M, et al (2014) The Thyroid Status of Children and Adolescents in Fukushima Prefecture Examined during 20–30 Months after the Fukushima Nuclear Power Plant Disaster: A Cross-Sectional, Observational Study. PLoS ONE 9(12): e113804 doi:10.1371/journal.pone.0113804 25474311 10.1371/journal.pone.0113804 PMC4256387

